# Commentary: How to have agency in a pandemic

**DOI:** 10.3389/frai.2023.1279759

**Published:** 2024-01-08

**Authors:** Rodney H. Jones

**Affiliations:** Department of English Language and Applied Linguistics, University of Reading, Reading, United Kingdom

**Keywords:** affect, agency, COVID-19, discourse, response-ability

## Introduction

The eight articles in this Research Topic touch upon the many disruptions to people's lives caused the COVID-19 pandemic, from the ways mandated lockdowns constrained their mobility and forced them to formulate new ways of interacting with friends and loved ones, to the new practices that they had to incorporate into their daily lives such as mask wearing and contract reporting, to the altered relations of power and (dis)trust that developed between citizens and their governments. They talk about how the very space they inhabited changed around them—cities becoming silent, the spaces in which they operated shrinking, and the space between bodies suddenly becoming something to be measured and monitored. They also discuss they ways time became distorted as the routines that people had previously used to order their movements through life were suddenly interrupted, and their ability to plan for the future was curtailed.

All of these social and material disruptions, as these articles illustrate, also involved disruptions in *discourse*: new terminology had to be learned, new conversational routines had to be mastered, new regulations had to be communicated and complied with, and new forms of storytelling had to be called upon to help people explain to themselves and to one another what they were going through. Closely related to these discursive disruptions, however, were more fundamental disruptions to *agency*. On the one hand, the new discursive regimes that developed around the pandemic, with their terminology and regulations and routines, played a major part in robbing people of their sense of agency. On the other hand, as their ability to control what was happening in their environments seemed to dwindle with each new media report and each new government policy—the words they used, the conversations they had, the ways they responded to official discourses, and the stories they told become even more central in helping them to maintain some sense of autonomy and authority over their affairs. The pandemic did not just transform the ways in which people affected and were affected by other people and things around them, but raised more fundamental questions about the very nature of action, autonomy and accountability, as well as questions about the role of discourse in making sense of and navigating a world of shifting power relations and shrinking possibilities. In this brief commentary I would like to explore the different perspectives on the relationship between discourse and agency reflected in these eight articles and what they can teach us as individuals and as societies about how to have (and not to have) agency during a pandemic.

Some of these articles address issues of agency explicitly. Robinson et al. (2023), for example, examine how agency the loss of agency was lexically and grammatically encoded in the way people talked about regulation; Wilding et al. (2023) show how older adults in isolation negotiated their loss of agency through their use of metaphors, and Cowie et al. (2022) describe the ways people coped with the disrupted relationship between structure and agency that came from forced immobility through the production of chronotopic discourse. In others, attention to the issue of agency is more implicit, though no less central, Tragel and Pikksaar (2022), for instance, focusing on how relationships of authority and solidarity were constructed in regulatory discourses about mask wearing, Bafort et al. (2023) addressing mediatized debates about personal freedom and privacy associated with COVID-19 telephone contact tracing, Kania (2022) discussing how practices of naming COVID-19 in media discourse revealed underlying ideological projects to assign responsibility for the pandemic to radicalized others, Giorgis et al. (2023) documenting the ways metaphors of warfare used by the governments functioned both as calls to action and constraints on agency in different countries, and Banga and Bellinzona (2023) exploring how municipal spaces became arenas in which negotiations among regulatory and transgressive discourses played out. In all of these treatments of the pandemic, discourse is presented as the primary means through which agency was claimed and constrained, power was exercised and resisted, and responsibility was assigned and denied. At the same time, across these different treatments of the pandemic, agency is not always conceptualized in exactly the same way. Sometimes the political dimensions of power and resistance are emphasized, sometimes psychological aspects of self- efficacy are the focus, and sometimes the ways agency emerged as an interactional accomplishment are highlighted.

Agency, of course, is itself a highly contested concept within the social sciences, with scholars debating whether it is necessarily “human, individual, collective, intentional, or conscious” (Ahearn, 2001, p. 130), arguing about the factors that amplify and constrain it such as privilege (Maxwell and Aggleton, 2013), material conditions (Kirchhoff, 2009), access to resources and other forms of capital (Bourdieu, 1977; Sewell, 1992), individual competencies (Bandura, 2006), or discursive regimes of knowledge/power (Foucault, 1995; Bleiker, 2003), and the degree to which it aligns with other concepts such as “freedom,” “control,” “rights,” and “responsibilities”. I will begin my discussion with Duranti's (2004, p. 453) “working definition”, which, although not entirely uncontroversial, covers most of the key dimensions of agency addressed in these papers:

Agency is here understood as the property of those entities (i) that have some degree of control over their own behavior, (ii) whose actions in the world affect other entities' (and sometimes their own), and (iii) whose actions are the object of evaluation (e.g., in terms of their responsibility for a given outcome).

What is useful about this definition is that it touches on agency as an *individual's* “capacity” to act (tying it to notions such as freedom and autonomy), as a *social* phenomenon whereby individuals affect and are affected by other entities (people, institutions, other organisms), and as the basis for the production of *accounts* regarding who or what is responsible for particular outcomes or states of affairs. Crucially, it is from such accounts that we come to understand how we got to where we are and imagine where we might go in the future. It is also from these accounts that we come to construct our worlds “moral” or “rational” places.

As a linguistic anthropologist, Duranti also provides a good starting point for understanding the relationship between language and agency. Language, he says, is related to agency in two ways. First, it is a tool for the *enactment* of agency. Simply by speaking, Duranti argues, we exercise agency, projecting our intentions out into the world. Agency is also inherent in the way we use language to divide up the word and create relationships between people and objects in it, the way we name things and frame situations. And, of course, as Austin (1976) has taught us, language is also one of the main tools we have at our disposal to *do* things—from directing others to act through commands and requests, to committing ourselves to action through promises, to actually changing reality through pronouncements of various sorts.

Just as important, though, is language's role in *representing* agency. Indeed, notions about if and how agency can be assigned to different entities in the world is encoded in our language, and, notably, different languages come with different opportunities for encoding agency. Language is also the means by which we make ourselves and others *accountable*, by which we attribute blame, take responsibility, claim rights, and perform all of the other evaluative work associated with agency.

It would, however, as Duranti points out, be a mistake to consider these two relationships between language and agency as separate. They are, in fact, mutually constitutive. “The enacting of agency”, he writes (2004, p. 454), “its coming into being—relies on and simultaneously affects the encoding—how human action is depicted through linguistic means”, a point that is made abundantly clear in a number of the papers in this collection, from the way the encoding of agency on public signs (see Tragel and Pikksaar, 2022; Banga and Bellinzona, 2023) provides people with the means to manage social relationships and enact or resist regulations, to the ways the encoding of agency in people's everyday talk can sometimes function as a means of reclaiming agency or challenging those who seek to constrain us (see Cowie et al., 2022; Robinson et al., 2023; Wilding et al., 2023).

A focus on language alone, however, is not sufficient to fully appreciate the complex, socially situated negotiations of agency described by the authors of these papers, most of whom align more with discourse analytical approaches in which agency is not just something that is encoded in language, and not just a matter of an *individual's* capacity to act, but rather is an interactional accomplishment that is as “intrinsically historical and situated” (Robinson et al., 2023) deeply embedded in social practices (Bourdieu, 1977; Giddens, 1984) and contingent on relationships of power, which are, in part, produced and reproduced through discourse (Foucault, 1995). This perspective is better captured by Ahearn (2001, p. 112, emphasis mine) more concise definition of agency as “the *socioculturally mediated* capacity to act”. It is this sociocultural mediation manifested in things like government policies, genres of interaction, linguistic landscapes, and life histories that these authors are particularly concerned with.

At the same time, there is also a way to read the findings of these studies through more post-human and new materialist perspectives in which agency is not enacted through the neat binary of “structure and agency” but rather through complex “flows of human and non-human vitality” (Gilmore, 2012). Such perspectives urge us to see agency as dynamically distributed among people, objects, technologies, institutions and organisms (such as viruses) (Latour, 2007), and newly emergent in every action and interaction (Barad, 2007). They also invite us to go beyond rational and representational concepts such as intentionality and governmentality and engage with agency more as a matter of *affect*, the immanent, transpersonal capacity for bodies to affect and be affected by one another (Massumi, 2002).

In what follows I will draw on all three of these perspectives on agency to explore what these papers have to teach us about “how to have agency in a pandemic”. In the next section I will consider what these papers tell us about how agency is encoded and enacted in language and discourse—through, for example, the grammatical structures and metaphors we use to talk about viruses and diseases. In the section after that I will explore how these papers formulate the relationship between structure and agency though their treatment of concepts such as power, regulation, resistance and responsibility. In the following section I will take up the ways these papers, often more implicitly than explicitly, offer insights into the more distributed and affective dimensions of agency. I will end by arguing that, while each of these perspectives on agency opens a valuable window on how people acted, reacted and were acted upon during the COVID-19 pandemic, they fail to provide a viable roadmap for “how to have agency” in the next pandemic in ways that more effectively address the tensions, conflicts and contradictions described in these papers. For this, I will argue, we need to turn to new conceptualizations of agency that are developing within education studies (see, e.g., Biesta, 2006; Ingold, 2017; Geerts, 2021) in which agency is less a matter of acting and more a matter of expanding the possibilities for action, less a matter of being and more a matter of becoming, and less a matter of “taking responsibility” and more a matter of increasing our capacity to respond moment by moment to situations and to those around us in ways that are open and present.

## Naming and framing

The dual role of language in both enacting and representing agency is particularly salient when it comes to talk of health and illness, especially where the forces that are causing illness are often invisible and/or contested. Pandemics are not “biomedical facts” so much as sets of “understandings, relationships, and actions that are shaped by diverse kinds of knowledge, experience, and power relations, and that are constantly in flux” (Brown, 1995, p. 37). This shaping takes place, according to Brown, through discourse—primarily thorough practices of “naming and framing”.

Naming is perhaps the most elemental way that humans seek to exercise agency over nature. By giving things names, we distinguish them from other things and make them concrete “objects” that can be analyzed, discussed, debated, and hopefully, controlled. But sometimes naming can create confusion and conflict rather than clarity, especially when the status of what we are trying to name is itself unclear. Often different names come refer to the same thing, or separate names need to be assigned to different dimensions of that thing. New diseases, especially when they reach epidemic proportions, are inevitably accompanied by what Banga and Bellinzona (2023) refer to as “terminological pandemics” or what Treichler (1999), writing about AIDS, called “epidemics of signification”, that spread as scientists, politicians, journalist and ordinary people try to make sense of the new malady and develop a language with which to talk about it.

The most important thing about naming, especially as it relates to agency, is that it is never ideologically neutral. Not only does the way we divide up the world and assign labels to the objects in it amplify and constrain possibilities for action, but naming is also the central process through which we assign *responsibility* (praise or blame) for actions that have occurred. In other words, naming is always to some degree a political act. This is the key point that Kania (2022) makes in her corpus-assisted analysis of the names used to refer to COVID-19 and the virus that causes it (technically SARS-CoV-2) in British newspapers. What is interesting, is first of all, the fact that the names associated with COVID that are considered “inappropriate” by the World Health Organization because they are thought to incite fear or hatred do so primarily though the way they directly or indirectly assign agency—terms such as “killer bug” or “deadly virus” assigning agency to the virus itself, and terms such as Wuhan virus or Chinese virus implying that responsibility lay with a certain group of people. Even more interesting is the way practices of naming can themselves become acts of provocation, the use of “inappropriate” names functioning as ways to attract attention, signal political affiliation, or hail certain kinds of audiences. Kania notes, for instance that “inappropriate” names were particularly prevalent in headlines, as well as in tabloid newspapers. Another obvious example is then President Trump's pointed use of the term “China virus” and attacks on those who called him out on it. Where agency is sometimes most powerfully enacted and encoded, then, is not in practices of naming themselves, but in metapragmatic discourse about naming (on the part of the WHO, politicians, and journalist). In Kania's data this can be seen in the way some journalists attribute “inappropriate” naming practices to others as a way of making them accountable, while others embrace “inappropriate” naming practices as a way to accuse those who negatively evaluate these practices of weakness or “political correctness”.

Of course, words do not exist in isolation. It is the way words are grammaticalized—that is, brought into relationships with other words—and the ways they are enmeshed in broader networks of associations, ideas, stories, and discourses, that make them such powerful tools for enacting and encoding agency. This is why Robinson et al. (2023) approach of “concept mapping” turns out to be such a useful way to interrogate the relationship between language and agency in the context of the pandemic. Their analysis of a corpus of 12 May Diaries from the Mass Observation Project reveals, perhaps not surprisingly, that REGULATION was a key concept in people's talk about COVID, manifested in their use of a cluster of interrelated words such as limitation, restriction, clampdown, freeze, timing, and coordination. The important thing, they point out, is not just how much people talked about REGULATION, but how REGULATION was grammaticalized in ways that reveal diarists' feelings of reduced agency. Examples of this include the objectification of actions through nominalizations (such as “recruitment *freeze*”), the use of passive voice (such as “the role *has been suspended*”), the use of agentless existential clauses (e.g., *there has been* no evidence of proper coordination), and the use of phrases (such as “complete uncertainty”) which lack reference to any particular agent or actor. When agents were named, they tended to be either politicians (e.g., Boris Johnson) or institutions (such as universities, large grocery suppliers). But even actions that could presumably be attributed to institutional actors such as the Government were often expressed in ways that hid responsibility for the action (e.g., the “easing of restrictions”). It is not so much that people constructed themselves as victims of other people (or entities) that were imposing restrictions on them, but rather that restrictions themselves seemed to take on “a life of their own” (Robinson et al., 2023). The key insight here is how the pandemic, for these particular diarists, and for people more generally, resulted in a pervasive “de-agentivation” of social actors (van Leeuwen, 2008, p. 23–74), a sense that nobody was in control of anything, which engendered a kind of collective gesture of surrender in the way people talked about the situation.

One of the most powerful ways that language (re)frames people's understanding and experience of agency is in the use of metaphor. Metaphorical language was so pervasive during the pandemic that it is touched upon, at least implicitly, in every one of these articles, Bafort et al. (2023), for instance, talking about how journalists discredited government responses to COVID by comparing them to failed responses to terrorist attacks, Kania (2022) discussing how different “inappropriate” names for the virus connected it to different domains of experience (e.g., animals and geography), and Banga and Bellinzona (2023) describing some of the visual metaphors that featured in the linguistic landscape of Italy during lockdowns. It is in the papers by Giorgis et al. and Wilding et al., however, that metaphorical language is taken up most explicitly and directly linked to issues of power, control and agency.

The prevalence of metaphors of war in the public discourse surrounding the pandemic, especially that emanating from official sources, has been widely studied (e.g., Panzeri et al., 2021; Semino, 2021; Benzi and Novarese, 2022), and these studies have found that the relationship between such metaphors and people's sense of agency can be complex. On the one hand, war metaphors can increase people's sense of collective agency by holding up the possibility of victory, while, on the other hand, they can also create feelings of fear and powerlessness and make people more willing to surrender their freedom and autonomy. One of the most problematic aspects of war metaphors is the way they discursively construct an “enemy” (the virus), onto which they impute a kind of malevolent intentionality. So, while talk of war can make people feel more “powerful”, it can also make the virus seem more powerful and threatening. Another problem is the inevitable slippage between the virus and people associated with it (such as those thought to be spreading it). Where Giorgis et al. add nuance to this literature is their cross-cultural approach, which shows that the ways war metaphors were used, and the ways they affected the agential landscape of the pandemic, differed in different political and cultural contexts. In Italy, for example, while early use of war metaphors by the government invoked past wars of liberation from Fascism, creating a sense of national unity, when the metaphor was taken to its extreme, with uniformed military patrolling the streets and a general appointed to manage vaccine logistics, memories of militarization during the Fascist period stoked public distrust. In Bulgaria, the politically motivated militarization of the pandemic by the government ended up being co-opted by anti-government forces and conspiracy theorist who mobilized war metaphors to resist restaurant closures and vaccination drives. Interestingly, war metaphors associated with the pandemic were not pervasive in the Ukraine, where an actual war was going on. These examples revel both how the use of war metaphors as a tool to consolidate power or mobilize the population can sometime have unexpected consequences, and the “potentially fuzzy boundary between the literal and metaphorical status of military references during the pandemic” (Semino, 2021).

While Giorgis et al. focus on the metaphorical language associated with the pandemic in official discourse, Wilding et al. address the way ordinary people in lockdown used metaphors to negotiate their sense of agency and to sometimes counteract the potentially disempowering effects of official metaphors. What is of particular interest here is not just the ways metaphorical language can shape power relations in the social and political spheres, but the way the metaphors we use can reveal something about our states of mind and the profound psychological effects exposure to metaphorical language can have on people feelings of self-efficacy (Bandura, 2006). Wilding et al. draw on the work of Charteris-Black (2021), who argues that container metaphors used to discuss isolation during the pandemic, and invasion metaphors used to characterize the virus, constituted a kind of “moral coercion” designed to engender feelings of resignation and disempowerment in the public. What Wilding et al. are able to show with their more qualitative exploration of the way older people subject to lockdown restrictions used metaphors is that, while much of their language exhibited a similar kind of personal “de-agentification” observed by Robnison et al.—participants portraying themselves at the mercy of agentive forces outside of their control such as the virus, time, and even their own emotions (see below), they also exhibited a resistance to using the metaphors that were prevalent in official discourses at the time and formulated alternate metaphorical frames in an attempt to reassert agency. One of these involved using metaphors associated with patterns and structure as a way to re-introduce feelings of control in their lives. Whereas for the diarists studied by Robinson et al., the concept REGULATION was associated with a loss of individual agency, for the participants in Wilding et al., REGULATION, in the form of *self-*regulation was precisely what allowed them to reassert agency, a finding which resonates with some psychological perspectives on agency which emphasize the ability to self-regulate as an essential ingredient in developing agency over other people and over situations (Bandura, 2006).

Finally, several of these papers note how people used language to frame their experiences of agency and, in some cases, to assert or reclaim agency, through the way they discursively constructed time and space in their talk and writing. Robinson et al., for example, discuss how diarists' narrativization of their experiences of the pandemic often exhibited fragmented portrayals of time, manifested, for instance in disconnected accounts of mundane events, discussions of hypothetical (uncertain) futures, and accounts in which the regulations themselves “became the new measure of time”. Similarly, Wilding et al. describe how their participants portrayed time as moving ahead of them and carrying or propelling them into the future rather than as something that they themselves moved through.

In contrast, the study by Cowie et al. (2022), also using diary data, paints a more positive picture, describing how people created different spatio-temporal frames in their narratives of the pandemic and used those frames to position themselves in relation to the situation they found themselves in. Central to their analysis is Bakhtin (1981) notion of the “chronotrope”, the way configurations of time and space are represented in discourse and how these representations come to be associated with particular social identities or “figures of personhood” (Agha, 2007). “[T]he most productive aspect of the chronotope concept” argues Blommaert (2015, p. 109, emphasis mine), both for the analysis of literary fiction and of sociolinguistic realities, is “its connection to historical and momentary *agency*” which enables “social and political worlds in which actions become dialogically meaningful, evaluated, and understandable in specific ways”. In their analysis of the ways people in the Edinburgh and the Lothian area of Scotland who were living alone represented their experiences of time-space before and after the lockdown, Cowie et al. found that different kinds of people produced different kinds of chronotopes. For international students, who before the lockdown lived rather regimented and restrained lives associated with their status as students and outsiders, the lockdown chronotrope was depicted as a space-time of change and opportunity which allowed them to re-negotiate their status as residents of the city. For retirees, the lockdown chronotope was also associated with increased agency and an enhanced ability to “keep busy”, as many social activities were suddenly accessible online. For men living close to the city center, however, the lockdown represented a loss of freedom and autonomy. These findings don't just remind us that the pandemic restrictions were not experienced by everyone as a loss of agency, but also how different ways of discursively framing restrictions can sometimes make available new kinds identities for social actors and, along with them, new possibilities for social action.

## Articulating structure

Many of the observations above regarding the encoding and enactment of agency in language paint a rather traditional (Western) picture of agents as autonomous, independent individuals seeking to maintain or increase their independence and autonomy in the face of restrictions placed on them. But that is only a partial picture of the way agency is portrayed in these articles. Along with this individualistic orientation toward agency, the authors, in various ways, also engage with the relational, dialogic emergence of agency in the context of social practices (Bourdieu, 1977). In this more practice oriented approach, agency is always enacted within the constraints of or against the backdrop of “structure” (Giddens, 1984), but the notion of structure is often ill-defined in discussions of structure and agency (Block, 2015), sometimes seen as an agentless, amorphous force, not so different from the way REGULATION is discursively constructed by the diarists in the paper by Robinson et al. In reality, the forces that constrain our agency are not just rules and regulations, but complex configurations of other agentive and non-agentive entities with whom we interact in various direct and indirect ways. Elder-Vass (2008) suggests three different dimensions of structure: *institutional structure*, which is comprised of institutions, organizations, broader “systems” of governing and exchange, along with the normative expectations they impose upon individuals and groups; *relational structure*, which is comprised of social relations with others, friends, family members, authority figures, and the kinds of rights and obligations that adhere to these relationships; and, *embodied structure*, which is comprised of the abilities and habits people develop that enable them to reproduce or resist institutional and relational structures. Block (2015, p. 20) adds to this list the structure imposed by the physical environment, in particular, “the spaces within which we are confined and within which we move” (which seems a particularly important addition in the context of thinking about structures around the COVID-19 pandemic). The way we discursively enact and encode agency is as much about how we engage in *dialogues* along these different dimensions of structure, and how we put these different dimensions of structure into dialogue with one another, than it is about asserting our individual freedom and autonomy or feeling “empowered”.

This interactional dimension of agency is seen in the ways the journalists in Kania's (2022) study formulate their naming practices in dialogue both with the norms established by the WHO and the practices of other journalists and politicians. It can be seen in the way the diarists in the study by Robinson et al. negotiate the limits of their physical environments, the dynamics of their workplaces, and their relationships with friends in order to get things done. And it can be seen in the different ways the different residents of Edinburgh experience and (re)frame institutional and relational structures in the study by Cowie et al.

In the context of these complex interactions, it is often not just the way people discursively construct agency, but the way they discursively construct structure—that is, which dimensions of structure that they choose to orient to—that can determine how they experience their capacity to take action. This was particularly evident during the pandemic when, for many, such as the Bulgarian conspiracy theorists discussed by Giorgis et al., the orientation was almost completely toward institutional structures—the machinations of a power hungry government and the scientific establishment—making *resistance* seem the only form of action available to them to enact agency. This particular orientation toward structure as chiefly institutional (and possibly authoritarian) was no doubt exasperated by the willingness of many governments to use the pandemic to stifle dissent and expand state powers, often under the banner of waging “war” on the virus (Giorgis et al., 2023). Many others, however, oriented more toward relational and environmental dimensions of structure, focusing more on their responsibilities toward friends and family members and the threat of the virus itself, mostly accepting the restrictions imposed by institutions and governments as necessary and reserving their ire for uncooperative fellow citizens who did not follow the rules. This did not necessarily make them less agentive; as Ahearn (2001) notes “agentive acts may also involve complicity with, accommodation to, or reinforcement of the status quo”.

Importantly, how people oriented toward structure and the kinds of negotiations they were able to have around agency were often dependent on their positions of privilege (Maxwell and Aggleton, 2013) or marginalization within their societies, determined by things like socioeconomic status, race, gender and age. The ability to “stay at home” or engage in “social distancing”, for example, was often as much a barometer of power and privilege as it was of “good citizenship” (Bennett, 2021). At the same time, as Cowie et al. note, sometimes it was those who entered the pandemic already accustomed to navigating restrictions (foreign students, pensioners) who were more able to adapt, whereas those who were accustomed to more freedom and autonomy (Scottish men) had trouble coping when their privileges were curtailed.

In most cases, people's negotiation of agency in the face of institutional restrictions did not take the form of direct negotiations with governments or institutions themselves, but rather were worked out at the level of interactions with individuals or other entities that took the role of mediating between the public and the government. Chief among these were commercial establishments, which were often put in the position of enforcing government regulations around things like mask wearing and social distancing, and the media (including social media platforms), which were often put in the position of explaining and interpreting government policy to the public as well as critiquing it, and of making determinations about what counted as “information” and what counted as “misinformation”.

The mediating role of commercial establishments in promulgating and enforcing government regulations can be seen most clearly in the paper by Tragel and Pikksaar, where they examine the ways authors of COVID-19 door signs in Estonia managed their relationships with customers through grammatically encoding markers of power and solidarity. This paper is also a good example of how the institutional dimensions of structure interacted in sometimes complex ways with relational dimensions of structure during the pandemic. As Tragel and Pikksaar observe, commercial establishments were often put in the awkward position of imposing restrictions on their customers' agency by, for instance, requiring them to wear a mask or produce a certificate of vaccination in line with government guidelines. This position was particularly difficult for small business owners who desperately depended for their income on their customers' goodwill. In communicating these restrictions on door signs, certain grammatical constructions, such as the use of the imperative mood and the second-person only (e.g., “Wear a mask and provide a COVID certificate!”) ran the risk of alienating customers by positioning them as subordinate and positioning the establishment as the authority who was imposing the restrictions rather than just enforcing them. To mitigate this risk and create more of a sense of solidarity with their customers, shopkeepers employed a range of linguistic strategies such as using self-directed language (first person pronouns) along with imperatives (e.g., “Dear guest, please wear a mask when entering our house”), avoiding imperatives altogether (e.g., “We ask for mask-wearing. Thanks!”), and portraying a party other than themselves (usually the government) as the source of authority (e.g., “Dear customer! Regarding the restrictions imposed by the government wearing a mask in the service station is mandatory”). What Tragel and Pikksaar demonstrate with their detailed analysis is how agency is not a simple matter of power and resistance, but rather something that usually emerges out of complex discursive negotiations among *multiple* parties with different goals. Understanding the mechanics of how these negotiations play out, they rightly point out, is essential for improving crisis communication.

In their mediating role between the public and authorities, commercial establishments also played a part in either promoting the policies of the government and the ideologies underpinning them, or in critiquing and resisting them, a fact that is amply attested to in Banga and Bellinzona's study of the linguistic landscape of Florence at different stages of the pandemic. In the early stages, they note, many shopkeepers used creative strategies (such as humor) to urge compliance with government guidelines and the make them seem more palatable. In doing so, they argue, commercial establishments also reproduced the ideological frames of unity, solidarity and patriotism that were being promoted by authorities. Later in the pandemic, however, as business struggled with the economic effects of restrictions and the public wearied of them, commercial signs began to adopt strategies such as sarcasm in order to subtly critique government guidelines as they were urging compliance with them.

The media, of course, played the most significant role in communicating government policies to the public and mediating negotiations of agency. In many contexts, of course, media outlets assumed the role of promulgating and legitimating information that came from the government and from mainstream medicine and science, and even alerting audiences to “fake news” and “unreliable sources of information”. There were also, of course, media (and social media) outlets that took a more skeptical stance toward official discourses and even provided a platform for conspiracy theorists. Most media outlets in western democracies, however, occupied a kind of uncomfortable middle ground between these two extremes, cognizant of their responsibilities to both disseminate essential information from authorities and to maintain their role as “watchdogs” against government and corporate malfeasance or disinformation. Attempts to achieve the latter goal were often, true to a long tradition in western journalism, framed in terms of debates about government encroachment on individual agency and autonomy and government accountability. These framings are evident in the study by Bafort et al., in which they compare media depictions of COVID-19 contact tracing to the interactions that actually occurred between contact tracers and members of the public. As they point out, contact tracing, in which citizens who have tested positive for SARS-CoV-2 were asked to report to authorities the names of people with whom they had come into contact during the time they were infectious, was a kind of “new genre” that many in the public were not familiar with, as well as a genre where issues of power, control and autonomy were particularly salient. What is interesting about Bafort et al.' analysis of actual contact tracing interactions and the policies and principles that informed the training of contact tracers, is how much attention was paid to mitigating effects on individual agency and to enacting egalitarian and empathetic interactions. In their analysis of media coverage of the program, however, they found that, rather than reporting accurately on what actually occurred in contact tracing interactions, journalists tended to focus on the inherent power imbalance of the enterprise and to invoke abstract, libertarian concerns about privacy and freedom. Not only was this discursive resistance to the policy misinformed, the authors argue, but journalists' readiness to frame contract tracing in terms of a structure-agency binary actually jeopardized public health.

## Distributed agency and affect

Above I examined how issues of agency were explored in these contributions through the lens of traditional frameworks like self-efficacy and practice theory. More recent treatments of agency in social science, however, have challenged the idea of agency as a property of human individuals or groups, suggesting instead that agency is distributed across networks of human and non-human entities. Among the most influential versions of this perspective is Latour's (2007) Actor Network Theory (ANT), which proposes that agency is not something that actors possess, but rather something they *perform* though the way they position themselves in relationship to other actors (both human and non-human). Another prominent view of agency that questions the idea of the unitary human agent is Barad's (2007), Agential Realism, which sees agency as something that emerges from the casual relationships between entangled phenomena (human and non-human, material and discursive), none of which have pre-existing ontologies. Agency arises when, through various material-discursive interventions, separations are enacted among these phenomena so they are made to seem distinct—what Barad refers to as “agential cuts”. In the more traditional views of agency which we have considered so far, agency is political insofar as it results from uneven distributions of power. But the political ramifications of post-human and new-materialist views of agency are even more profound, since the very act of separating out entities as able to “have” agency is an essentially ontological exercise which determines not just who or what has power, but also who or what “matters” or is excluded from mattering. At the same time, there is also perhaps, more room for hope within these perspectives. Because the capacity to act is not fixed within the structure-agency binary, but rather dynamically performed across agential fields, more possibilities are opened up not just for “reclaiming” agency, but for reconfiguring social worlds (Introna, 2014).

Although none of these articles engage explicitly with this understanding of agency, there are hints of it in for example, the ways the diarists in the study by Robinson et al. portray themselves as navigating and even (re)-configuring assemblages of regulations, objects (such as groceries), people and institutions in order to get things done, the way the diarists in the study by Cowie et al. engage with the material and affective dimensions of their environments, the way the contract tracers in the study by Balfort et al. operate as parts of assemblages of individuals, institutions, discourses (such as scripts) and technologies (telephones), and in the ways the elderly respondents in Wilding et al. attribute agency to the virus and even to their own emotions. Although, in the context of more traditional ideas about human agency, such attributions of agency to non-human entities are seen as disempowering, from the point of view of the approaches described in this section, they might be regarded not only as ontologically more accurate but also as potentially creating space for people to enact agency *in concert* with other entities rather than seeing it as a “zero-sum game”—something that people “have”, and so, something that can be taken away from them. Gilmore (2012, p. 91), in her discourse analysis of diaries of people experiencing pain suggests that [p]osthumanism offers a way to rethink agency, enabling a focus on how, through their speech and writing, people are able to “re-craft or re-image their symbolic and material body and its borders” in the context of what she calls “agency without mastery”.

One aspect of these papers where these more post-human perspectives on agency might be explored further is the way they engage with the notion of *affect*. Scholars in the field of affect studies also see agency as emergent and distributed. What they add to this conversation is the assertion that the best way to understand how agency emerges in the (intra)relationship among entities is through the lens of “affect”, which they see as “bodily capacities to affect and be affected…to engage, and to connect” (Clough, 2007, p. 2; see also Spinoza, 1985; Deleuze and Guattari, 1987). From this perspective, agency is inseparable from the ways bodies attract and repel each other, inseparable from desire and fear, from anger and joy, and from grief and hope. All of these feelings have the capacity to reconfigure agential fields, bringing us closer to some entities and pushing others away. One thinks, for example, of the dramatic ways the participants in the study by Wilding et al. describe their emotions as seemingly independent entities that seem to “creep up on them” and pull them in different directions, or of the complex and sometimes contradictory emotions the diarists in Robinson et al. express about regulations, or of the way the international students in Cowie et al. “feel” the city of Edinburgh differently during lockdown. One also thinks of the way affect can be deployed by others to undermine agency by generating fear or hatred, such as when metaphors of war or labels such as “China virus” become prominent features of the discursive environment (Kania, 2022; Giorgis et al., 2023).

Without a doubt, the paper that engages most fully with notions of distributed agency and affect is the study of the pandemic landscapes of Florence by Banga and Bellinzona, in which they join in a long tradition of considering the affective dimensions of physical environments, from the “affective turn” in Linguistic Landscape studies which they mention (Milani and Richardson, 2021), to other work using concepts such as “affective atmospheres” (Anderson, 2009) and “affective geographies” (Jones et al., forthcoming; O'Grady, 2018). In their description of the streets of Florence at different stages of the pandemic, Banga and Bellinzona show not just how the physical environment became a canvas upon which the collective “shock” of residents was expressed, but also came to function itself as an agent, “structuring the affective affordances and positions of individuals and groups (Wee and Goh, 2020, p. 139, cited in Banga and Bellinzona, 2023)”. Rather than just seeing agency as enabled and constrained by institutional and relational structures, there is a sense in the descriptions they provide of the streets of Florence of agency emerging out of “atmospheres” which are collectively formed from the countless “affective-discursive practices” (Wetherell, 2015 p. 160) of the city's residents, atmospheres that have concrete material consequences on people's behavior and sense of self-efficacy, either creating space for acts of solidarity and charity or of overwhelming people with feelings of rancor and despair. This version of agency as an *ecological* phenomenon contingent on the momentary and dynamic coming together of “bodies, subjectivities, relations, histories, and contexts” (Wetherell, 2015, p. 160) is radically different from the view of agency presented in the other papers in this collection, and in some ways more hopeful, suggesting that it is sometimes in moments when people put aside the drive for individual autonomy and control and orient instead to affectively aligning themselves with others—friends, strangers, enemies—and with their material circumstances, that possibilities for coordinated action, collective responsibility and genuine empathy arise.

## Conclusion: agency as response-ability

So, what can we take from these papers that can teach us how to have agency in a pandemic, a question that seems particularly important given that we didn't seem to do a very good job of it last time around? Sadly, much of our inability to take action against the virus—so much of the suffering and death that we witnessed—was not the result of the virus itself, but the result our failure to figure out how to take collective action, a failure seen on the level of nations, institutions and communities. So much of our time and energy seemed to be spent defending borders, assigning blame, and asserting “rights”, and many of the policies pursued by governments seemed designed not just to isolate us physically, but to isolate us morally, clothing neoliberal discourses of privatized risk and individual responsibility (Lupton, 2013) in collective gestures of solidarity, like simultaneously clapping for underpaid and overworked healthcare workers. Attempts to critique the restrictions that were being placed upon us by governments often veered between the extremes of unquestioning compliance and radical libertarianism, and the ways individuals responded to these restrictions became more a matter of protecting political or ideological territories than of protecting public health. So much time and energy was spent separating out those who were doing the right thing from those who were not that we forgot to ask what “doing the right thing” really means, and what kinds of material conditions, social relationships, moral codes, medical knowledge and embodied desires are necessary to enable us to know what the right thing to do is.

Perhaps the main thing that these contributions teach us about how to have agency in a pandemic is that language matters, that the way we talk about things—in official discourse, in the media, and in our individual interactions with one another—can have profound effects on our ability take individual and collective action. The ways that we linguistically assign agency and responsibility to different entities through things like metaphors and transitivity, as well as the ways we use language to label different kinds of actions and different kinds of people as right or wrong, friends or enemies, helps to constitute the psychological and social environments in which actual actions are carried out. The way we use language can exasperate feelings of distrust, isolation and disempowerment, but it can also provide opportunities for strengthening connections with others and spaces for reimagining and creativity reconfiguring our realities. This came out particularly strongly in the articles which featured the voices of ordinary people telling stories about their lives in the context of diaries or interviews. As Cowie et al. intimate, just the action of writing a diary entry for an audience of the future is acknowledgment of responsibility and a gesture of hope. They quote De Fina's (2021, p. 60) assertion that “through narratives, participants bring to bear in their present interactions worlds and historical moments that belong to different geographical and temporal scales” and in so doing “create new understandings of reality and also new patterns of social interaction”. In this regard, it seems that the questions we need to be asking about the relationship between language and agency need to go beyond questions about how agency is encoded and enacted in language to questions like those suggested by Pratt (2018, p. 24, emphasis mine) in a discussion of the role of language in socio-cultural creativity: “What gives utterances the ability to generate *courage*? To *move* people from one belief to another, to compel *action*? How does speech *emancipate* and generate new futures? What qualities give speech the world-making, subject-producing, transformative powers we see exhibited every day?”

Another thing I think we can learn from these contributions is how possibilities for action are not static, but arise out of inter (and intra)-actions with other people and with our environments. Agency does not have to be seen as a “zero-sum game” in which individuals and institutions vie for power, and it is not always enacted in terms of resistance to structure. Engaging with more relational and post-human approaches to agency can help us generate new perspectives on how people understand and talk about the different forces (both human and non-human) that come to constitute the agential fields in which they operate. They can also sensitize us to the fact that the course of pandemics are not determined by the autonomous actions of individuals and governments but by the ways individuals and government position themselves in relationship with a host of other actors. As Geerts (2021, p. 158), reminds us, a pandemic is a “multilayered more-than-human crisis that requires a holistic, but non-totalizing, approach”.

To really understand how to have agency during a pandemic, however, and to avoid the mistakes we made in the last one, requires that we come to grips not just with how agency intersects with issues of courage, creativity and empowerment, but also how it intersects with notions of collective responsibility and empathy. Here we might take inspiration from work in education studies (e.g., Biesta, 2006; Ingold, 2017; Geerts, 2021) which challenges the idea that agency is prior to and determinative of action. “[J]ust because not everything happens according to one's own volition does not mean that someone else is in charge, or that agency is more widely distributed” writes Ingold (2017, p. 24). Rather, possibilities for action are continually “forming and transforming from within the action itself”, so that instead of talking about agency, we should talk about “agencing”. In order to see possibilities for action as they emerge moment by moment, however, requires a shift in perspective away from notions of individual “responsibility”—which seek to concentrate power and to situate blame—to notions of “response-ability”, the ability to respond to (rather than just react to) the circumstances that arise in our social and material worlds. This applies both to the ability of governments and institutions to flexibly respond to quickly changing health crises, as well as to individuals' ability to respond to the needs, capacities, fears, and desires of others, to search for opportunities for connection even in contexts where our normal ways of connecting are constrained. As Biesta (2006, p. 64) puts it, “what is done, what needs to be done, and what only I can do, is to respond to the stranger, to be responsive and responsible to what the stranger asks from me”. Biesta insists that the whole point of education is to help people to cultivate the capacity to respond and be responded to, not just to question and answer, but to also to recognize and be present to others, to identify common ground and articulate possibilities for collective action. One source of inadequacy in most approaches to health education is their focus on *self-*efficacy rather than relational efficacy—their preoccupation with telling people how to *behave* rather than how to respond. Similarly, one source of inadequacy in the approaches to understanding language and agency reviewed here is their focus on speaking rather than listening—their preoccupation with the discursive strategies that people use to claim agency for themselves rather than the discursive strategies they use to take action with others.

By the way it moved among us, the virus revealed the precarity of the human community in which the default for many seemed to be to react rather than respond, to close boarders rather than to open doors, and to seek ways to capitalize on others' suffering rather than to relieve it (Butler, 2020). At the same time, it also revealed—through the countless individual and collective gestures of care and selflessness it provoked—gestures that courageously resisted the default—our capacity to respond, and it reminded us that sometimes true agency is less about freedom and more about generosity, less about mastery over our environment and more about learning from it.

## Author contributions

RJ: Writing—original draft.
